# Ocular findings in Fabry disease in Colombian patients

**DOI:** 10.7705/biomedica.3841

**Published:** 2019-09-01

**Authors:** Katherine Rothstein, Jubby M. Gálvez, Ángela M. Gutiérrez, Laura Rico, Eveling Criollo, Alejandra de-la-Torre

**Affiliations:** 1 Grupo de Investigación en Oftalmología, Escuela Superior de Oftalmología, Instituto Barraquer de América, Bogotá, D.C., Colombia Grupo de Investigación en Oftalmología Escuela Superior de Oftalmología Instituto Barraquer de América BogotáD.C Colombia; 2 Grupo de investigación Geniuros, Escuela de Medicina y Ciencias de la Salud, Universidad del Rosario, Bogotá, D.C., Colombia Universidad del Rosario Grupo de investigación Geniuros Escuela de Medicina y Ciencias de la Salud Universidad del Rosario BogotáD.C Colombia; 3 Grupo de Investigacion en Neurociencias, Escuela de Medicina y Ciencias de la Salud, Universidad del Rosario, Bogotá, D.C., Colombia Universidad del Rosario Grupo de Investigacion en Neurociencias Escuela de Medicina y Ciencias de la Salud Universidad del Rosario BogotáD.C Colombia

**Keywords:** Fabry disease, alpha-Galactosidase, lysosomal storage diseases, corneal opacity, retinal vessels, lens capsule, crystalline., enfermedad de Fabry, alfa-galactosidasa, enfermedades por almacenamiento lisosómico, opacidad de la córnea, vasos retinianos, cápsula del cristalino

## Abstract

Fabry disease is a rare X-linked disorder caused by an alpha-galactosidase enzyme deficiency, which leads to a progressive lysosomal glycosphingolipids accumulation, mainly globotriaosylceramide, in multiple organism tissues including the eye. This case series describes the first ophthalmological Colombian report of Fabry disease highlighting the importance of ocular signs as markers of the disease, useful in diagnosis and treatment to avoid long-term complications that lead to a morbi-mortality increment. We describe five cases of Fabry disease from Bogotá, Colombia, including a complete clinical history, ophthalmologic, optometric examination, and photographs. We found that all patients had refractive defects and that in all cases corneal verticillata pattern was found. Four patients presented with posterior capsule lens brown-beige deposits and four patients had conjunctival and retinal tortuous vessels. A complete ophthalmologic examination is important for prompt diagnosis, which is key to starting a multidisciplinary treatment and reducing morbi-mortality.

## RESUMEN

La enfermedad de Fabry es un raro trastorno ligado al cromosoma X causado por deficiencia de la enzima alfa-galactosidasa y la consiguiente y progresiva acumulación lisosómica de glucoesfingolípidos, especialmente la globotriaosilceramida, en múltiples tejidos del organismo, incluido el ojo. En este reporte se presenta la primera serie de casos de manifestaciones oculares de la enfermedad de Fabry en Colombia, resaltando la importancia de los signos oculares como ayuda para el diagnóstico temprano. Se presentan cinco casos de la enfermedad en Bogotá y se da cuenta de las historias clínicas y los exámenes oftalmológicos y de optometría, y se incluyen fotografías. En todos los pacientes se hallaron errores de refracción y se evidenció el patrón de córnea verticillata. Cuatro pacientes presentaban depósitos de color café y castaño claro en la cápsula posterior del cristalino, y cuatro tenían tortuosidad vascular conjuntival y retiniana. El examen oftalmológico completo es importante para hacer un diagnóstico oportuno con el fin de iniciar el tratamiento multidisciplinario y reducir la morbimortalidad.

Fabry disease is a rare X-linked disorder caused by alpha-galactosidase deficiency which leads to a progressive lysosomal glycosphingolipids accumulation, mainly globotriaosylceramide (Gb3) ^1^ in endothelial and smooth muscle cells of several organs vessels including the eye ^1^^-^^4^.

Clinical manifestations begin during infancy and adolescence and include intermittent acroparesthesias ^5^, limbs chronic pain ^3^^,^^6^, “Fabry crises” ^1^^,^^5^, angiokeratomas ^5^^,^^6^, hypo-anhidrosis ^5^, gastrointestinal and cardiac abnormalities ^1^^,^^5^^,^^7^^,^^8^, cerebrovascular attacks, and ocular abnormalities ^2^^,^^9^^,^^10^.

The disease occurs primarily in the cornea, conjunctiva, lens, and retina. Ophthalmological features of Fabry disease have been described not only in the anterior segment but also in the posterior pole of the eye. Anterior segment manifestations are aneurysmal vessels in the bulbar conjunctiva, cornea verticillata, and opacification of the lens. In the posterior segment, the most common features are vessel tortuosity and retinal vascular occlusions ^1^.

Early diagnosis is important to start a prompt enzyme replacement therapy ^11^^,^^12^ and to prevent new ocular complications and irreversible systemic organ damage, although enzyme replacement therapy does not reverse the established ocular manifestations of Fabry disease ^2^. 

The purpose of this case series is to present the ocular findings of Fabry disease in a Colombian series of cases to improve diagnosis, remission, and early treatment of these patients, avoiding long-term complications that can increase morbidity and mortality. 

## Case series

Four male patients diagnosed with Fabry disease and a carrier woman, all from Bogotá, Colombia, were referred to a reference ophthalmologic center for evaluating their ophthalmological features. The diagnosis was previously made by geneticists considering the clinical findings by testing for deficient α-galactosidase A (α-GAL) enzyme activity and confirmed by DNA analysis of the α-GAL A gene. Four patients belonged to the same family: Three brothers and the mother identified as patients 1, 2, 3, and 4 (table 1).

**Table 1:** Demographic, systemic and ophthalmological findings in five Colombian patients with Fabry disease

**Case**	**Gender**	**Age**	**Family history of Fabry disease**	**Neuropathy**	Organs affected besides eye at the moment of the ophthalmic evaluation	**Ametropy**	**Ambliopy**	**Cornea verticillata**	**Lens opacity**	**Conjuctival vascular tortuosity**	**Retinal tortuous vessels**	**Lacrimal abnormality**
1	Male	14	Yes	No	No	Mixed astigmatism	Yes	Yes	Yes	Yes	Yes	No
2	Male	19	Yes	Yes	No	Simple myopic astigmatism	Yes	Yes	Yes	Yes	Yes	No
3	Female	38	Yes	No	No	Simple myopic astigmatism	No	Yes	No	No	No	No
4	Male	17	Yes	Yes	No	Mixed astigmatism	Yes	Yes	Yes	Yes	Yes	No
5	Male	43	No	No	Kidney	Simple myopic astigmatism/ mixed astigmatism	Yes	Yes	Yes	Yes	Yes	Yes

All of the patients (or relatives/guardians) signed the assent and/or informed consent. A complete clinical record with family and personal history, optometric-ophthalmological examination and ocular photographic register of anterior and posterior segments of the eye were completed (table 1).

**Figure 1:**
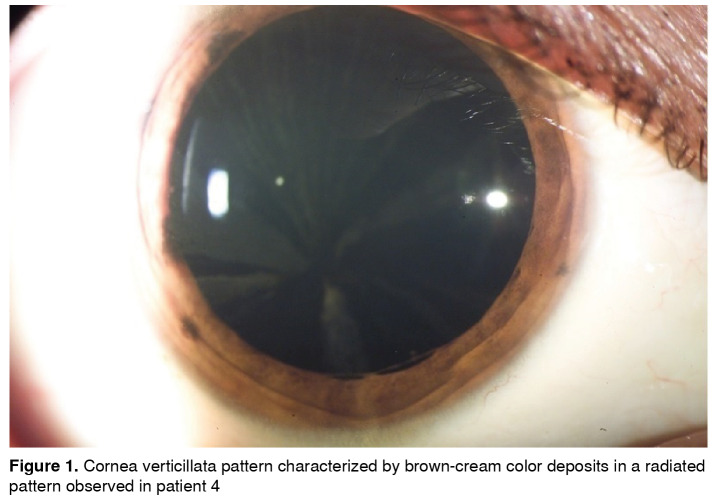
Cornea verticillata pattern characterized by brown-cream color deposits in a radiated pattern observed in patient 4

**Figure 2:**
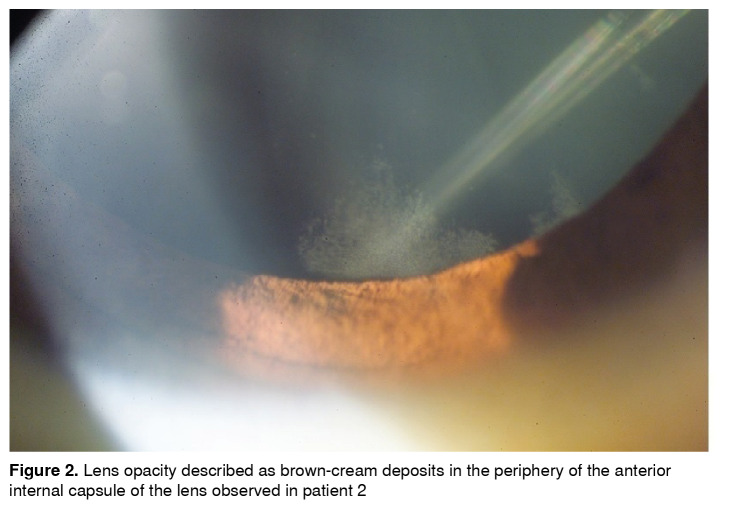
Lens opacity described as brown-cream deposits in the periphery of the anterior internal capsule of the lens observed in patient 2

Refractive defects were found in all patients as composed myopic astigmatism, three with mixed astigmatism and amblyopia. They all had punctate brown-cream corneal deposits with a central corneal radiated pattern, which in advanced cases generated a verticillata pattern (figure 1). Four patients had a wedge shape brown-cream deposit on the anterior internal lens capsule periphery, predominantly inferior (figure 2). All of them except for the carrier female presented with conjunctival and retinal vessels tortuosity (table 1). 

Four of the five patients presented the ‘classic variant’ of Fabry disease and only one presented the ‘kidney variant’ with severe renal compromise requiring kidney transplantation (table 1). The starting time of enzyme replacement therapy was not specified in the clinical charts of the patients.

## Discussion

Ophthalmological findings are common in Fabry disease compromising various ocular structures ^1^^,^^2^^,^^8^^,^^9^^,^^13^. In our case series, one of the most typical ocular findings was cornea verticillata pattern found in all of the patients; four out of five presented with lens opacity and with tortuosity and dilatation of retinal and conjunctival vessels. Although four of five presented with tortuous vessels, none of them developed retinal venous-arterial occlusions and none presented with optic disc edema.

Corneal epithelial-subepithelial deposits are the most important ocular manifestation ^7^ presenting in more than 95% of affected males and 88% of carrier females. The injury starts during infancy as a diffuse cloud, which progresses to cornea verticillata, characterized by radial, linear, symmetric, and bilateral cream to brown-gold color deposits ^14^.

Cornea verticillata is the most distinctive finding in females. All males in our case series presented cornea verticillata, as well as other ocular findings such as conjunctiva, lens, and retinal alterations. In contrast, the only woman among our patients presented cornea verticillata without the aforementioned findings. Cornea verticillata is rare in subjects without Fabry disease and, therefore, this finding has high diagnostic sensitivity and specificity, often related to disease severity ^15^.

Other causes of cornea verticillata are amiodarone prolonged therapy, aminoquinoline, atovaquone, subconjunctival gentamicin, gold salts, ibuprofen, indomethacin, naproxen, phenothiazine, tamoxifen, multiple myeloma, and environmental exposure to silica dust^16^. All pathologies and/ or drugs that can produce similar findings were discarded in the patients.

Vascular conjunctival abnormalities include vessel dilatation, tortuosity, and aneurysms, which are more common in the inferior bulbar conjunctiva ^1^^,^^14^. Vascular tortuosity is related with disease severity ^17^. Similarly, except for the female, all of our patients presented with conjunctival abnormalities.

In the literature, lens opacities are present in 70% of males and 35% of carrier females. Posterior opacities are the most common in both genders. Linear white opacities reveal the Gb3 epithelium deposits through the posterior suture lines known as Fabry cataracts, the only ocular diagnostic criteria ^1^. Anterior cataract, seen in 31% of males and 5% of females, is usually bilateral and wedge-shaped, with a variable density. Helix form, granular cream color deposits are found in the anterior capsule ^4^^,^^14^. According to this, lens opacities were observed in all four males of our case series. Figure 2 shows the linear-white opacities compatible with Fabry cataract.

The most common retinal finding is vascular tortuosity ^1^, found in 77% of males and 19% of carrier females. A narrowing of small vessels can cause a vascular-occlusive phenomenon secondary to Gb3 accumulation. Therefore, Fabry disease should be considered when a young patient presents a retinal or peripapillary vascular-occlusive event. Several patients have visual loss caused by retinal central artery-vein occlusion, ischemic optic neuropathy, optic atrophy, retinal ischemia or choroid perfusion defects ^1^. Although retinal vascular tortuosity was observed in four of our male patients, there were no vascular-occlusive events.

Increased blind-spot has been reported without an afferent disc defect or dyschromatopsia. Other findings include myopia, nerve fibers myelination, and disc edema ^14^^,^^18^. None of our patients had optic nerve alterations. All of them presented with refractive errors consistent with astigmatism and three of them with myopic astigmatism.

Because most ocular findings do not compromise visual acuity, patients rarely consult ophthalmologists. Nevertheless, when a characteristic Fabry sign is observed, it should be suspected and referred for a complete screening, especially when the patient is young, as in such cases ophthalmologists may identify an early-stage disease before complications appear. Ocular signs can act as markers of the disease with diagnostic and prognostic implications. As the eye is an external organ easily investigated with minimally invasive technologies, it may be useful for monitoring the natural history of Fabry disease and its response to enzyme replacement therapy ^17^.

All the patients in this series were referred to our clinic with the diagnosis of Fabry disease for ophthalmological examination and ocular findings were present in all of them.

The clinical systemic presentation is heterogeneous and the signs and symptoms change with the age of the patient due to the slow progressiveness of the disease ^17^^,^^19^. Three of our patients were male siblings under 20 years of age who presented with acroparesthesia, neuropathic pain, joint and abdominal pain crisis, and dyshidrosis. They did not report cardiac, respiratory symptoms or stroke, probably because such symptoms often appear at a later age ^19^. Patient 5, the oldest in the case series (43 yearsold), had a renal variant, several of the symptoms already mentioned, such as pain attacks, and was under enzymatic treatment. Additionally, he was taking immunosuppressant medications for post-transplant management.

An exhaustive ophthalmological assessment offers an important opportunity for early diagnosis. Any patient with corneal opacity, cornea verticillata or Fabry cataract in association with retinal vessels tortuosity, conjunctival telangiectasia or lens opacities should have a detailed evaluation and appraisal of family history. If there is a high risk of Fabry disease, the patient should be referred to a geneticist for evaluation and genetic counseling. If it is confirmed, the patient can be treated and multidisciplinary management should be started before irreversible complications appear ^19^. The treatment does not depend only on the diagnosis; rather, since each patient is different, the treatment pathway should be adjusted and managed according to the individual.

Greater awareness of Fabry disease among optometrists and ophthalmologists could reduce diagnostic delays and decrease early mortality of these patients.
